# Production and characterization of pulp and paper from flax straw

**DOI:** 10.1038/s41598-024-74096-y

**Published:** 2024-10-16

**Authors:** Tsiye Tekleyohanis Hailemariam, Belay Woldeyes

**Affiliations:** 1 Chemical Engineering, Debre Berhan University, Debre Berhan, Ethiopia; 2 Chemical Engineering, Addis Ababa University, Addis Ababa, Ethiopia

**Keywords:** Flax straw, Kraft pulping, Morphological analysis, Pulp and paper, Plant sciences, Environmental sciences, Engineering

## Abstract

Flax (*Linum usitatissimum*) is a bast fiber plant known for its long fibers, making it an excellent source of pulp for paper production. In Ethiopia, flax is primarily cultivated for oil, with the residual straw utilized for papermaking. This study focuses on pulping flax straw using the Kraft process and investigates its chemical composition, proximate analysis, and morphological properties. The proximate analysis revealed an ash content of 4.13% and moisture content of 11%. Chemical composition analysis showed cellulose at 51.34%, hemicellulose at 25.20%, lignin at 14.12%, ash at 4.13%, and extractives at 5.21%. The morphological properties included a fiber length of 1.41 mm, diameter of 16.78 μm, lumen width of 9.45 μm, and cell wall thickness of 3.77 μm. Flax straw exhibited an acceptable Runkel ratio (0.8) and flexibility coefficient (56.32), placing it within the range of non-wood fibers. SEM analysis of the pulp’s morphology was conducted to assess fiber structure, including the presence of cracks. Pulp quality and length are directly linked to paper strength. Various pulping conditions were studied using a full-factorial design, with optimum conditions being 10% alkaline, 131.74°C, and 120 min of cooking time, yielding a pulp with a Kappa number of 10.45 and a yield of 40.56%. The resulting paper demonstrated standard tensile, tearing, and burst strengths, indicating that flax straw is a promising raw material for paper production.

## Introduction

Flax (*Linum usitatissimum L*.), an annual plant predominantly cultivated in temperate regions, has significant potential within the Ethiopian agricultural sector^1^. Ethiopia’s vast agricultural landscape, where flax is widely grown, generates substantial amounts of flax straw as a byproduct. Traditionally, this flax straw has been underutilized, often discarded as waste. However, with the increasing global demand for sustainable and environmentally friendly raw materials, flax straw presents a promising lignocellulosic alternative for pulp and paper production^2^ .

The Ethiopian economy stands to benefit considerably from the valorization of flax straw. By integrating flax straw into the pulp and paper industry, Ethiopia could not only reduce reliance on imported pulp but also address critical environmental concerns associated with deforestation and resource depletion. This approach aligns with global trends, where non-wood fibers, particularly agricultural residues like flax straw, are increasingly recognized for their ecological advantages and potential to produce high-quality paper^3^.

Papermaking has a long history, dating back to its origins in China nearly 2,000 years ago, where non-wood materials were primarily used. Even today, in countries like China and India, a significant proportion of paper production relies on agricultural waste and non-wood plants, highlighting the viability of these materials. Despite the historical precedence and environmental benefits, the use of non-wood fibers in global pulp and paper production remains limited, accounting for only a small fraction of the raw materials used^4^.

In Ethiopia, the demand for paper is rising rapidly, driven by the needs of the education sector and other industries. To meet this demand sustainably, it is imperative to explore alternative raw materials like flax straw. This research aims to optimize the pulping conditions such as time, temperature, and active alkali concentration to maximize the yield and quality of pulp derived from Ethiopian flax straw. By doing so, this study seeks to boost the local flax straw valorization, contributing to both economic growth and environmental sustainability.

In conclusion, the utilization of flax straw in the Ethiopian pulp and paper industry offers a strategic opportunity to enhance the country’s economic and environmental resilience. This research will pave the way for the optimized use of flax straw, positioning it as a valuable resource in Ethiopia’s industrial landscape.

## Materials and methods

### Materials

Raw material treatment was conducted by sieve, and manual scan for removing unwanted material from pure flax straw^5^ at Addis Ababa University School of Chemical and Bio-Engineering laboratory (Addis Ababa, Ethiopia). Raw material, flax straw from Shewa agricultural research Centre was collected.

#### Sample preparation

Flax straw was cut into an average length of 2 cm^3^using the cutter and manual cutting in the Mechanical unit operation lab. Then the sample was ready for physicochemical characterization and pulping such as morphological^6^, analysis proximate^7^and chemical composition^8^ of flax straw.

### Physicochemical characterization of flax straw

#### Moisture content

The prepared flax straw was made free of moisture in an oven at 105±3°C overnight^9^and continue to stay in the oven until the constant weight of the prepared flax straw before cooking of pulp^10^. Then the moisture content was calculated by using the following formula at every two hours’ interval until a constant weight of flax straw was obtained^11^.

1 $$\:Moisture\:content\:\left(\%\right)=\frac{{m}_{1}-{m}_{2}}{{m}_{1}}\times100$$

$\:{m}_{1}$ = Mass of flax straw before drying

$\:{m}_{2}$ = Mass of flax straw after drying

#### Ash content

The ash content of the residual lignin samples was determined under Tappi Standard T 211 om-93 and T 211 om-02^12^. Approximately 5 g^13^was burned in a muffle furnace at 525°C^14^. A separate test sample was used to find the moisture content of the specimen^15^. The output weights of ash and moisture level in the sample was used to calculate the percentage ash present at 525°C^16^on a moisture-free sample basis by the relationship shown below^17,18^.

2 $$\:Ash\left(\%\right)=\frac{{m}_{1}\times\:100}{{m}_{2}}$$

Where:

$\:{\text{m}}_{1}$: is the weight of ash in grams

$\:\:{\text{m}}_{2}$: is the weight of the moisture-free test sample in grams. The weight of curicble subtract from both.

#### Extractives

According to^19^5 g of dried raw flax straw was loaded into the cellulose thimble and put within the Soxhlet extractor set up, 400 mL of acetone^20^was used as a solvent for extraction. Residence times for the boiling and rising stages were carefully adjusted to 80°C^21^and 25 min^22^respectively on the heating mantle for a 4 h run period^23^. After extraction, the sample was air-dried at room temperature overnight^24^. The constant weight of the extracted material was achieved in a convection oven at 105°C^25^. The %(w/w) of the extractives content was evaluated as the difference in weight between the raw extractive-laden flax straw and extractives-free flax^26^.

3 $$\:Extractives\left(\%\right)=\frac{{m}_{3}-{m}_{2}}{{m}_{1}}\times\:100$$

Where:

$\:{m}_{1}$ : Oven dry weight of sample (g)

$\:{m}_{2}$ : Oven-dry weight of flask (g)

$\:{m}_{3}$: Oven-dry weight of extract and flask (g)

### Chemical composition

#### The lignin content of flax straw

According to^19^and Tappi T 222 cm-99, 3 g^27^ of dried extracted raw flax straw was weighed in glass test tubes and 30 mL of 72% H_2_SO_4_^28^was added. The sample was kept at room temperature for 2 h with carefully shaking at 30 min intervals to allow for complete hydrolysis^29^. After the initial hydrolysis, 84 mL of distilled water was added^30^. The second step of hydrolysis was made to occur in an autoclave for 1 h at 121°C^31^. The slurry was then cooled at room temperature. Hydrolyzates were filtered through vacuum using a filtering crucible^32^. The acid-insoluble lignin was determined by drying the residues at 105°C^33^and accounting for ash by incinerating the hydrolyzed samples at 575°C in a muffle furnace^34^. The acid-soluble lignin fraction was determined by measuring the absorbance of the acid hydrolyzed samples at 320 nm in the spectrophotometer and used standard curve for acid-soluble lignin concentration^11^.

4 $$\:lignin\:content\:of\:flax\:straw=Acid\:soluble\:lignin+acid\:insoluble\:lignin$$

#### Hemicelluloses

According to^19^ 6 g of extracted dried flax straw was transferred into a 500 mL Erlenmeyer flask. 300 mL of 1000 mol/m^3^NaOH was added^35^. The mixture was boiled for 3.5 h with distilled water^36^. It was filtered after cooling through vacuum filtration and washed until neutral pH. The residue was dried to a constant weight at 105°C in a convection oven^37^. The difference between the sample weight before and after this treatment is the hemicellulose content (%w/w) of dry flax straw^38^. Weighed until the variation between two successive measurements were not greater than 0.005 and record as w_2_^39^.

5 $$\:Hemicellulose\left(\%\right)=\frac{{w}_{2}}{{w}_{1}}\times\:100$$

Where:

$\:{w}_{1}$: - is the oven-dried extractive free sample (g)

$\:{w}_{2}$: - is the weight of oven-dried hemicellulose (g)

#### Cellulose

According to^19^ the sample was dried at 105°C until a constant weight was achieved, then approximately 2 g of the dried sample were weighed. For holocellulose extraction, the sample was placed in a beaker with 80 mL distilled water, 1 mL acetic acid, and 1 gram sodium chlorite, and the mixture was heated to 70°C on a hot plate, stirring occasionally. The temperature was maintained, and additional acetic acid and sodium chlorite were added every hour for 4 h. After cooling, the mixture was filtered and the residue washed until neutral pH. For alpha-cellulose isolation, the residue was treated with 17.5% sodium hydroxide, stirred, filtered, and washed with 8.3% sodium hydroxide solution, followed by distilled water and 1% acetic acid. The residue was then dried at 105 °C and weighed.

6 $$\:cellulose\,content\left(\%\right)=(\frac{weight\:of\:dried\:alpha\:cellulose\:residue}{intial\:weight\:of\:the\:sample\:})\times100$$

### Morphological characteristics of flax straw

In the morphological analysis of flax straw including fiber length^41^, fiber diameter^21^, lumen width^22^, and cell wall thickness^3^. For fiber length determination in the flax straw, a small amount of flax straw was taken, macerated with 50 ml of HNO_3_, 67%^42^and distilled water with a ratio of 1:1^43^until the fiber separated in the solution and boiled in a water bath 100±2°C for 20 min^16^and wash with distilled water^38,44^.

For fiber diameter, lumen width and cell wall thickness determination, cross-sections were cut into small pieces manually. This cross-section was stained with a 1:1 aniline sulphate glycerin mixture to enhance cell wall visibility (cell walls retain a characteristic yellowish color). Three derived values were also calculated using fiber dimensions: slenderness ratio as fiber length /fiber diameter, flexibility coefficient as fiber lumen width/fiber diameter) *100, and Runkel ratio as (2* cell wall thickness /lumen width). The results were compared with flax, agricultural waste and other biomass according to^45^.

### Pulp production process

Pulping of chipped flax straw was achieved using the chemical pulping method (Kraft pulping). About 50 g of the raw material (oven-dry weight) was loaded into a 1000mL conical flask which was then placed in an autoclave. The study utilized a statistical model to evaluate the effects of three independent variables time, temperature, and active alkali on two dependent parameters: yield and kappa number. The liquor-to-biomass ratio was maintained constant at 9:1 throughout the experiments. The cooking conditions varied in terms of time (30 to 120 min), temperature (140°C to 170°C), and active alkali concentration (10–20%). Pulp yield and kappa number were measured, and the results were analyzed to determine the optimal conditions for maximizing yield and minimizing kappa number, considering the constant liquor-to-biomass ratio^46^.

#### Pulp bleaching

Flax straw pulps were submitted to an oxidative delignification process using hydrogen peroxide. This bleaching process was done in three stages, using hydrogen peroxide during the two initials, and only a sodium hydroxide solution in the last one. Initially, pulp slurry of 10% consistency was mixed with the corresponding amount of 5% H_2_O_2_and stirred at 80°C for 120 min. This procedure was performed two consecutive times. In the third and last bleaching stage, the pulp was mixed with a 0.25 M NaOH aqueous solution inconsistency of 10% and stirred at 70°C for 60 min. Subsequently, the bleached pulp was washed with excess distilled water to remove the residual alkali and the pH value of the sample was adjusted to 7 and dried at room temperature until approximately 10% moisture content^16^.

### Kappa number determination

The kappa number test was used to estimate the amount of lignin by measuring the.

oxidant demand of the pulp. Kappa numbers were performed on air-dried pulp samples, which was dried on a heated balance to acquire their oven-dried weight. The pulp was then treated with potassium permanganate (KMnO_4_) under TAPPI standard^17,34,47,48^.

### Surface morphological observation

The bleached flax straw pulp was examined under a Scanning Electron Microscope (SEM) to study its fiber morphological properties according to^49^ Scanning electron microscopy was performed to observe the morphology and surface structure by scanning the ruptured surface of pulp from flax straw. It was used to study the effects of various pulping condition and bleaching(H_2_O_2_) on the morphology of the samples. The operation principle for SEM to cross-sectional morphology of the sample was done under vacuum at an accelerating voltage of 2500 kV and x1806 magnification and also at different magnification. All the samples were firstly mounted on the surface of carbon tapes, loading on the top of aluminum stubs. The dirt was cleaned by the air jet and the samples were coated by a fine layer of gold with 20 mA for 2 min to avoid charge.

### FTIR analysis of pulp from flax straw

Fourier Transform Infrared Spectroscopy was used to investigate changes that occur in the chemical structure of pulp from flax straw after was investigated using Fourier transform infrared spectroscopy (FTIR) equipped with origin JASCO. This technique was used to manipulate structural changes in samples and to examine the changes in functional groups induced by various treatments as a result of chemical modification by the identification of the functional group). Where the spectra are performed at room temperature in the range of 400 to 4000 cm^−1^ with the resolution of change X cm^−1^and a total of 3736 points for the sample according to^50^.

### Refining condition of pulp from flax straw

The refining conditions of pulp from flax straw significantly influence its quality and performance. Key parameters include refining time, which optimally ranges from 5 to 30 min, refining temperature, typically maintained at 90°C, and pressure, set at approximately 1.5 MPa. Additionally, the consistency of the pulp during refining plays a critical role, with effective results observed at around 2 to 4% consistency. The application of chemical treatments, such as esterication, further enhances fiber deconstruction and improves mechanical strength. These optimized conditions are essential for maximizing the yield and quality of flax straw pulp for various industrial applications.

### Paper hand making

**Fig. 1:**
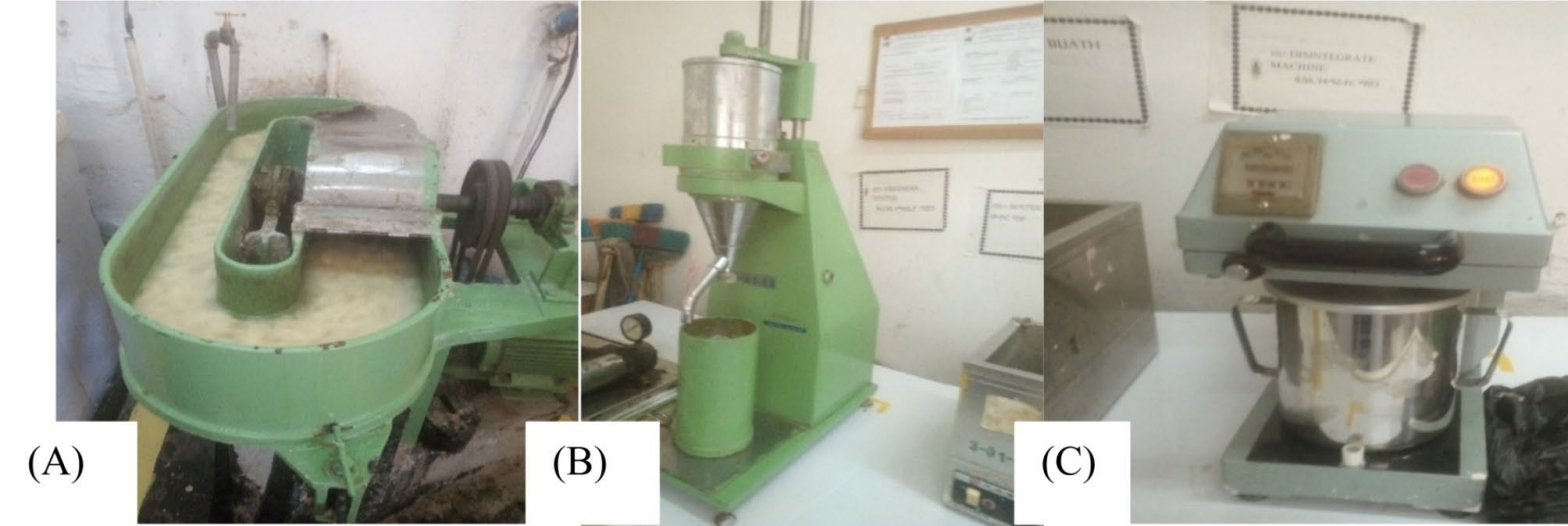
Paper hand-sheet making from flax straw **A**): Beating Machine **B**): Freeness Tester **C**): Pulper.

Paper sheet making from flax pulp according to Tappi standard^51^. Once the sheets shall prepare two stages of pressing were followed by applying 0.4 MPa pressure for two minutes by pressing machine in Fig. 1A pulp from flax straw was milled and n Fig. 1B check paper modification, dehydration, control properties of paper (strength, smoothness, opacity and brightness) and check the fiber quality, process control and consistency, and predicting paper making performance in Fig. 1C. Then the stock was removed from the press and attached to the drying plates to dry by oven at 100°C for 45 min according to Wonji pulp and paper factory laboratory manual and TAPPi standard^29^.

### Properties of paper

Properties of paper including tensile strength, bursting and tearing strength were tested according to Tappi standard in Wonji factory Ethiopia.

### Bursting index

The test specimens were first prepared as 10 cm$\:\times\:$10 cm sheet and clamped in the tester tightly with bursting strength tester of model PN-BSM600 and the maximum reading pointer was set to zero position. The pump motor was then started and the pumping system and the test piece then wait to burst. The maximum read pointer was then recorded and allowed to rest gently to zero position and the broken sample was removed. Instrument reading = kg/cm^2^according to Wonji laboratory manual, Tappi standards and^37^.

### Tear index

The pendulum was raised to its initial position and 4 pieces of specimens (65*7.5 cm) was clamped. According to^53^silt in the specimens were then made by completely pressed down the knife rocker arm. The pendulum was then stopped by quickly pressing down the releaser and the pendulum was broken softly after oscillation to the right. The scare value was record. The average of the reading was then calculated and multiplied with the factor of the pendulum. So the product was equal to the tearing strength in milinewton (mN) according to^52 and 53^.

### Tensile strength

The result of paper from flax straw was checked tensile strength using tensile strength tester after the sheet specimens were cut into 15±0.1 mm wide by 230 mm long test pieces, it was then placed in the clamps of Lorentzern & Wettre tensile strength tester by making sure that any slacks were so eliminated. Any touch of the test area between the clamps with the fingers was avoided. All readings were then recorded except for test pieces that broke with in 2 mm of clamming line according to Tappi (T 494 om-01).

### Control properties of paper characterization from flax straw

Opacity, Brightness, Strength control properties was investigated according to^54^.

## Results and discussion

### Raw material characterization

The chemical compositions of flax straws are a great indication of pulp and paper production. The properties of flax fibers are listed in Table 1. As seen, the cellulose of flax straw somewhat is larger than wheat straw, rice straw and bamboo. From the below table, the current study and past research, the cellulose content of flax straw is higher than the other three biomasses (wheat straw, rice straw, and bamboo) except Enset. The cellulose content of flax straw is 51.43% greater than the other three (wheat straw, rice straw, and bamboo) crops except for Enset which has 59.6% in Table 1^55^.

The holocellulose content of flax straw is 74.85% much higher than other biomass or agricultural waste which is wheat straw, rice straw, and bamboo^56^. This indicated that flax straw was preferable for the production of pulp as input for the production of paper. The above experimental data the higher cellulose content used for the production of pulp with its quality.

**Table 1:** Chemical composition of flax straw and some other non-wood material with dry weight basis.

Biomass	Holocellulose (%)	Cellulose (%)	Hemicelluloses (%)	Lignin (%)	Ash (%)	Extractives (%)
Flax straw (current work)	76.54	51.34	25.20	14.12	4.13	5.21
Wheat straw [57]	74.50	47.51	27.00	15.30	4.70	5.50
Rice straw [39]	64.00	44.00	20.00	19.00	9.80	7.20
Bamboo [56]	66.28	46.00	20.28	22.17	1.10	11.55
Enset [55]	87.87	59.60	28.27	8.22	3.80	1.11

### Morphological analysis of flax straw

**Table 2:** Morphological analysis of flax straw and some other Non-wood material reported in the literature. a; Current study b 57; c39; d 56

Parameters	Flax straw ^a^	Wheat straw ^b^	Cotton Stalk ^c^	Bamboo^d^
Length (L), mm	1.41	1.21	0.83	2.30
Diameter (D), µm	16.78	17.20	24.38	15.1
Lumen width (d), µm	9.45	8.40	15.65	6.90
Cell wall thickness (W), µm	3.77	3.80	4.37	4.17
Slenderness ratio (L/D)	84.03	70.34	34.04	152.3
Flexibility coefficient, (d/D) *100	56.32	48.8	64.20	45.69
Runkel ratio, (2 W/d)	0.80	0.9 0	0.54	1.21
Fiber, %		39.80	45.5	50

As shown in Table 2 fiber dimensions (including fiber length, fiber diameter, lumen width, and wall thickness) are essential parameters of lignocellulosic materials. These parameters are closely associated with various structural, physical, and chemical properties of the plant. So from Table 2the current student flax straw length (1.41 mm) is preferable to wheat straw (1.21 mm)^57^and cotton stalk (0.83 mm)^39^. Based on this result flax straws are also very good indicators to decide the material suitability for different end products. They affect much wood-product processing, like the drying process, resistance to cutting and machining and pulpwood quality. Fiber dimensions are also related to different pulp quality indices like Runkel ratio, slenderness ratio, rigidity coefficient and flexibility coefficient. As a result of the experiment, flax straw has a more flexibility coefficient than wheat straw (48.8%) and bamboo (45.69%)^56^.

Fiber length has a positive correlation with burst strength, tensile strength and tear strength. Long fiber lengths are preferable for the production of good-quality of paper. As shown in the Table 2 Long fibers give a more drainable and less uniform sheet structure. Thin cell walls positively affect the flexibility, burst and tensile strength of paper based on these properties. The standard value for this ratio is one, satisfactory pulp strength is usually obtained when the Runkel ratio is below the standard value. A low Runkel ratio means a thin fiber wall and larger fiber lumen width in Table 2. A thin fiber wall is desirable for high-quality, dense and well-formed paper. Paper manufactured from thick-walled fibers will be bulk with a coarse surface. Moreover, large lumen size positively affects the beating of pulp, which involves the penetration of liquid into spaces within the fiber. Thus, fiber with a high Runkel ratio value will be stiff, less flexible and will form bulkier paper of low bounded area. The other important parameter is the slenderness ratio. If the value of the slenderness ratio is less than 70 the fibers are not used for good-quality pulp and paper production. The strength properties of paper such as tensile strength, bursting strength and tearing strength are highly affected by the individual fibers that are cross-linked together in the paper sheet. The degree of fiber cross-linked depends on the flexibility and compressibility of individual fibers in the pulp from the straw. The coefficient of flexibility, usually expressed in percentage, is derived from the ratio of lumen width to its fiber diameter.

### Pulp yield and kappa number

The yield and kappa number are the responses of the experiment conducted on the production of pulp from flax straw at different variables and levels, as shown in Table 3. The experimental variables temperature, time, and active alkali are each specified at three levels in Table 3 below. Each independent variable influences the experimental responses, including the kappa number and the yield of pulp produced from flax straw.

**Table 3:** Full experimental design matrix and response.

Std	Run order	Blocks	Time (min)	Temperature (℃)	Active alkaline (%)	Response	
						Yield (%)	Kappa number
27	1	Block 1	120	150	20	29.78	5.74
25	2	Block 1	60	150	20	37.98	9.42
1	3	Block 1	60	130	10	45.5	23
7	4	Block 1	60	150	10	41	11.39
26	5	Block 1	90	150	20	32	6.04
19	6	Block 1	60	130	20	42.78	19.18
2	7	Block 1	90	130	10	43	15.55
20	8	Block 1	90	130	20	37.75	13.01
17	9	Block 1	90	150	15	33.11	8.3
3	10	Block 1	120	130	10	42.1	10.39
14	11	Block 1	90	140	15	37.6	9.45
23	12	Block 1	90	140	20	35.01	7.89
16	13	Block 1	60	150	15	39.35	10.39
12	14	Block 1	120	130	15	40.13	9.64
10	15	Block 1	60	130	15	44	21.2
24	16	Block 1	120	140	20	32.95	4.75
22	17	Block 1	60	140	20	41.01	10.86
9	18	Block 1	120	150	10	34.34	8.34
11	19	Block 1	90	130	15	39.59	14
18	20	Block 1	120	150	15	31.09	6.4
5	21	Block 1	90	140	10	38.76	11.2
13	22	Block 1	60	140	15	42.13	11.98
21	23	Block 1	120	130	20	38.03	6.77
8	24	Block 1	90	150	10	35.07	9.25
6	25	Block 1	120	140	10	37.55	8
4	26	Block 1	60	140	10	43	13.89
15	27	Block 1	120	140	15	37.25	6.1

### Scanning electron microscopy (SEM) analysis of bleached pulp from flax straw

**Fig. 2:**
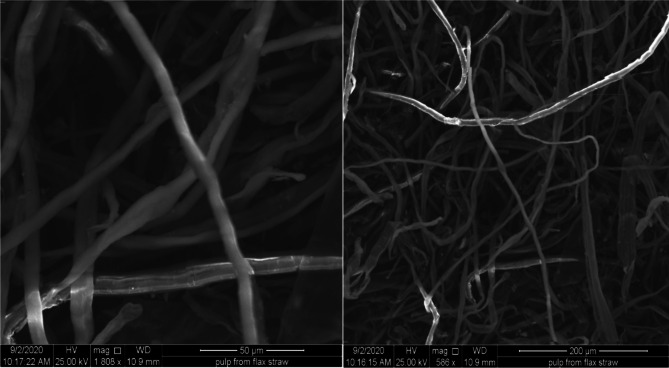
SEM analysis of pulp fiber from flax straw.

Scanning electron microscopy (SEM) analysis of the bleached pulp produced from flax straw. The SEM analyses were conducted on pulp with a yield of 40.56% and a Kappa number of 10.45. These values were obtained under the following conditions: a cooking time of 120 min, a temperature of 131.42 °C, and an active alkali concentration of 10%. Fibers were magnified at 200X and 50X are shown in Fig. 2. The analysis reveals the structure and arrangement of the fiber bundles inside the pulp fiber^49^. However, the strength of the fiber can be explained according to the order and formation of the fiber pattern. The pulp fibers exhibit a closely packed alignment, and their structure demonstrates a high degree of crystallinity, as observed through SEM analysis. This shows a higher fiber content as well as long fibers in the sample than the other species. This fiber structure could increase the fiber’s mechanical properties and the quality of the paper produced. Moreover, the compactness and arrangement of fibers play an important role in the quality of the produced paper besides the other parameters such as cellulose content in the non-wood materials^58^. As the pulp fiber as shown in Fig. 2 highly cross-linked and aligned to each other, the final output of the paper SEM structure is highly cross-linked giving high strength of paper.

### FTIR analysis of bleached pulp from flax straw

**Fig. 3:**
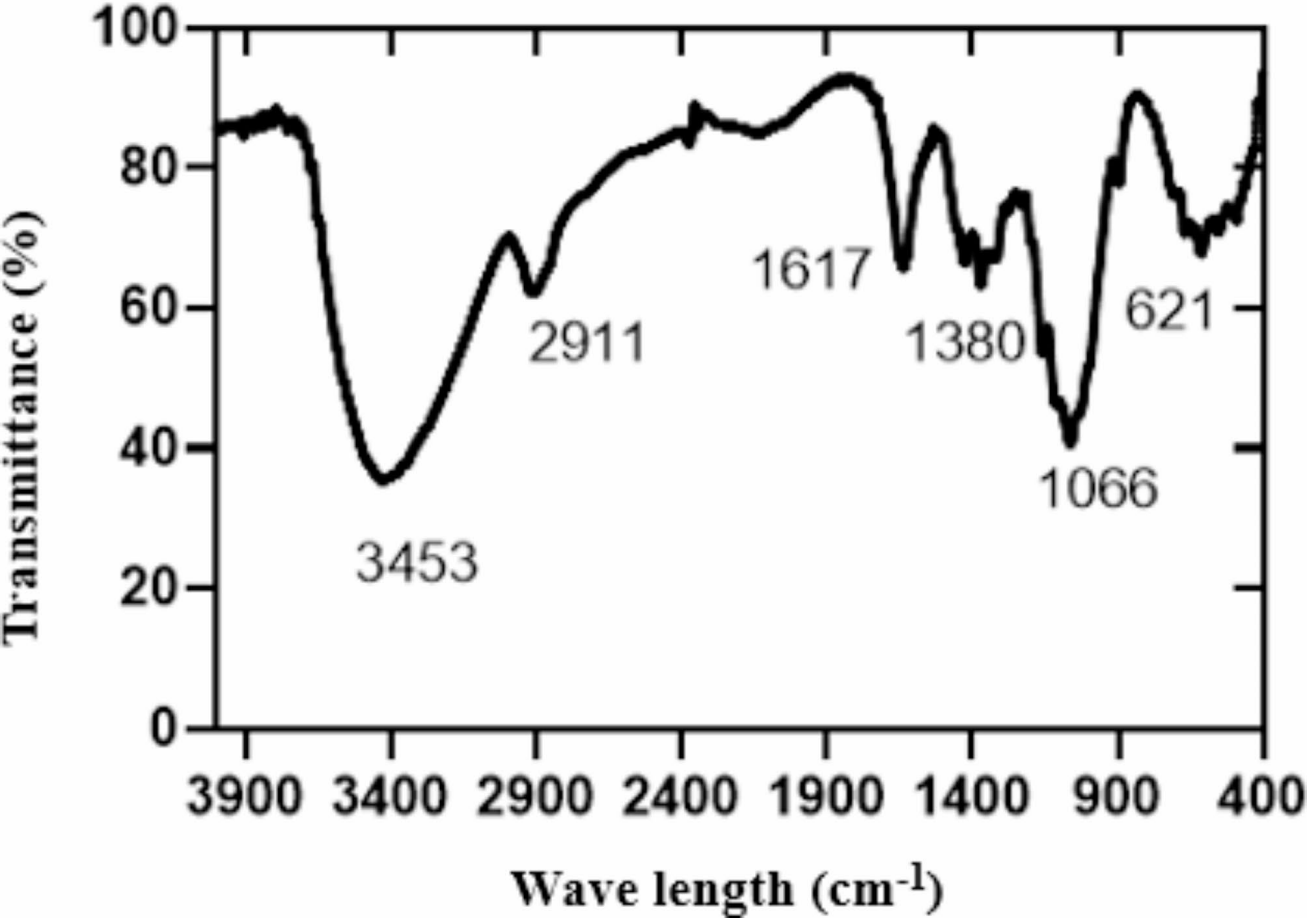
FTIR analysis of bleached pulp from flax straw.

FTIR spectra of cellulose fibers from flax straw pulp are 13 peaks as shown in Fig. 3. The FTIR analyses were conducted on pulp with a yield of 40.56% and a Kappa number of 10.45. These values were obtained under the following conditions: a cooking time of 120 min, a temperature of 131.42 °C, and an active alkaline concentration of 10%. The absorption bands are observed in two wave number regions of 3500 –2900 cm^−1^ and 1617 − 400 cm^−1^. The presence of peaks on the spectra of cellulose samples coming from flax pulp corresponds to bands of microcrystalline cellulose of pulp from flax straw as shown in Fig. 3, Identification of the absorption peaks in each wavenumber is following. The observed peaks in the wave number range of 3500 –2900 cm^−1^are characteristic of the stretching vibration of O-H and C-H bonds in polysaccharides^59^. The broad peak at 3453 cm^−1^ is characteristic of the stretching vibration of the hydroxyl group in polysaccharides. This peak includes also inter- and intra-molecular hydrogen bond vibrations in cellulose. The band at 2911 cm^−1^ is attributed to CH stretching vibration of all hydrocarbon constituents in polysaccharides. Typical bands assigned to cellulose were observed in the region of 1617 –700 cm^−1^. The peaks located at 1617 cm^−1^ correspond to the vibration of water molecules absorbed in cellulose. The absorption bands at 1380, 1230, 1066 cm^−1^ and 900 cm^−1^ belong to stretching and bending vibrations of -CH_2_ and -CH, -OH and C-O bonds in cellulose. The band at around 1420–1430 cm^−1^ is associated with the amount of the crystalline structure of the cellulose, while the band at 700 cm^−1^is assigned to the amorphous region in cellulose^60^. FTIR was used to detect the presence of lignin in cellulose samples. Lignin was characterized absorption bands, particularly in the regions around 1500–1600 cm⁻¹ (aromatic C = C stretching) and 1700–1750 cm⁻¹ (carbonyl stretching). The presence of these peaks in the FTIR spectrum would indicate that lignin remains in the sample.

### Experimental analysis and adequacy check for full factorial models

The effect of temperature, time, and active alkaline on the Kappa number and yield of pulp has been mainly an effect by the quality of pulp. Then here, the resulting data is going to be analyzed to determine the significant factors of the experimental work by using DESIGN-EXPERT 13 software. Pulp yield and Kappa number obtained from laboratory experiments results were input to the software.

Pulp yield obtained from laboratory experiment results was Analyzed by a design expert and obtained the general yield formula, model type, F value, P-value, R^2^ and determine the significant effect of the factor on the yield of pulp.

**Table 4:** Analysis of variance (ANOVA) for the response pulp yield on flax straw. Source: Current Work from design expert.

Source	Sum of Squares	DF	Mean of Square	F- value	Prob > F	
Model	451.74	6	75.21	228.78	< 0.0001	Significant
A	159.19	1	159.19	484.21	< 0.0001	
B	194.44	1	194.44	591.42	< 0.0001	
C	60.61	1	60.61	184.36	< 0.0001	
A^2^	24.25	1	24.25	73.77	< 0.0001	
AB	10.27	1	10.27	31.23	< 0.0001	
AC	2.52	1	2.52	7.67	0.0118	
Residual	6.12	20	0.36			
Cor total	457.86	26				

The Model F-value of 228.78 in the Table 4implies the model is significant. There is only a 0.01% chance that an F-value this large could occur due to noise. P-values less than 0.0500 indicate model terms are significant^25^. In this case A, B, C, AB, AC, A² are significant model terms. Values greater than 0.1000 indicate the model terms are not significant^58^. If there are many insignificant model terms (not counting those required to support hierarchy), model reduction may improve model^61^.

**Table 5:** The value of R- Square on the yield of pulp under modified model.  Source: Current Work from design expert.

Std. Dev.	0.57	*R*-Squared	0.9856
Mean	38.22	Adj R-Squared	0.9813
C.V.	1.5	Pred R-Squared	0.9771
PRESS	10.49	Adeq Precision	55.458

In Table 5 the mean value and standard deviation above 95% for yield of pulp from the flax straw. This implies all variable significant impact of the product response (Yield).

Final Equation in Terms of Actual Factors:

7 $$\:Yield=+72.43667-0.023741\text{*}\:\text{A}-0.051167\,\text{*}\,\text{B}-0.092\,\text{*}\,\text{C}-0.003\,\text{*}\,\text{A}2-003\,\text{*}\text{A}\,\text{*}\,\text{B}-0.003\,\text{*}\text{A}\,\text{*}\text{C}$$

Where:

A: Cooking time.

B: Temperature.

C: Active alkaline.

### Model summary statistics in pulp kappa number

Under experimental investigation easily determined the optimum parameter or pulping condition, develop the design equation, determine the value of R-square, the interaction effect of the variable on the Kappa number of pulp and develop the modified design equation finally find out the best pulping condition with minimum Kappa number at an optimum yield at minimum cost and energy.

**Table 6:** Model summary statistics in pulp Kappa Number.  Source: Current Work from design expert.

Source	Sum of squares	DF	Mean square	F value	*P* value	
Mean	3160.74	1	3160.74			
Linear	461.07	3	153.69	37.5	< 0.0001	
2FI	56.17	3	18.72	9.83	0.0003	
Quadratic	30.49	3	10.16	22.74	< 0.0001	Suggested
Cubic	3.96	7	0.57	1.56	0.2537	Aliased
Residual	3.64	10	0.36			
Total	3716.07	27	137.63			

A quadratic model is recommended with 3 DF, 10.16 mean square and less than 0.0001 P- value as Table 6 in the model summary statistics in pulp Kappa number.

**Table 7:** Analysis of variance (ANOVA) for the response pulp Kappa number of flax straw.  Source: Current Work from design expert .

Source	Sum of Square	DF	Mean of Square	F- value	*P*-value	
Model	546.09	5	109.22	248.21	< 0.0001	Significant
A	236.02	1	236.02	536.4	< 0.0001	
B	183.49	1	183.49	417	< 0.0001	
C	41.56	1	41.56	94.44	< 0.0001	
B^2^	29.29	1	29.29	66.57	< 0.0001	
AB	55.73	1	55.73	126.65	< 0.0001	
Residual	9.24	21	0.44			
Cor Total	555.33	26				

There is only a 0.01% chance that a “Model F-value” this large could occur due to noise. Values of “Prob > F” less than 0.0500 indicate model terms are significant. In this case, A, B, C, B^2^, and AB are significant model terms. Values greater than 0.1000 indicate the model terms are not significant.

The Model F-value of 248.21 implies the model is significant in Table 7. There is only a 0.01% chance that a “Model F-value” this large could occur due to noise. All the models in terms of the pulping condition have a value of p above 0.1. This means the model is significant and the pulping parameter has significance on the Kappa number. Based on the F value in the Table 8 cooking time highly affect the value of the Kappa number.

**Table 8:** Value of R- Square on the Kappa number of pulp.  Source: Current Work from design expert.

Std. Dev.	0.66	*R*-Squared	0.9834
Mean	10.82	Adj R-Squared	0.9794
C.V.	6.13	Pred R-Squared	0.9715
PRESS	15.8	Adeq Precision	57.046

In Table 8 the mean value and standard deviation above 90% for Kappa number of pulp. This implies all variable significant impact of the product response (Kappa number).

Final Equation in Terms of Actual Factors:

8 $$\:Kappa\:number=+593.02833-1.12637*A-7.15222\,*\,B-0.3038\,*\,C+0.022098\,*\,\text{B}2+7.1833E003*AB$$

Where:

A: Cooking time.

B: Temperature.

C: Active alkaline.

### Effect of an individual factor on yield and kappa number

**Fig. 4:**
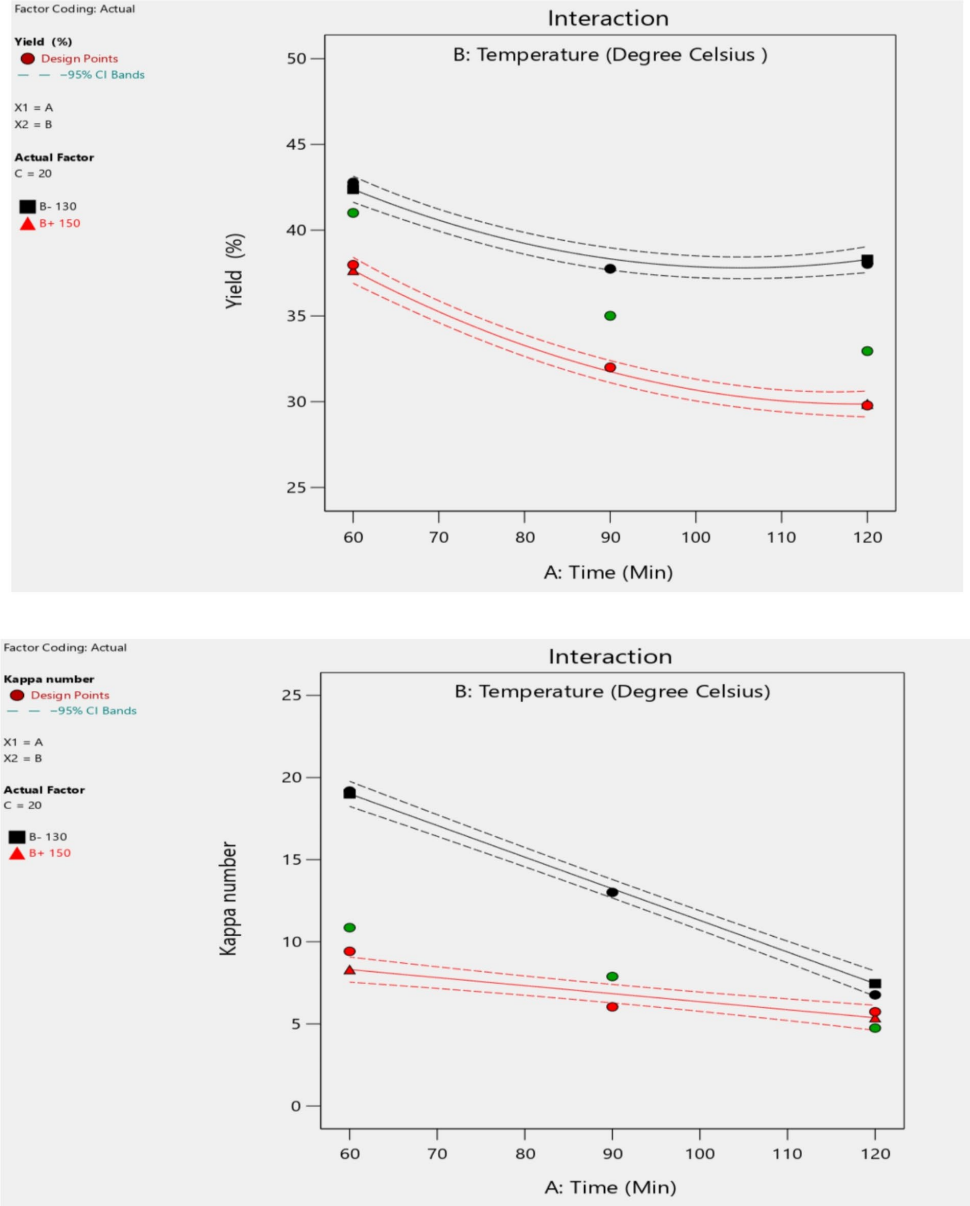
Effects of individual factors.

The most crucial diagnostic is a normal probability plot of the residuals as shown in Fig. 4. The actual vs. predicted data points are almost linear. Although the highlighted run does differ more from its predicted value than any other, there is no cause for alarm due to it being within the red control limits.

### Diagnostics statistics

**Fig. 5:**
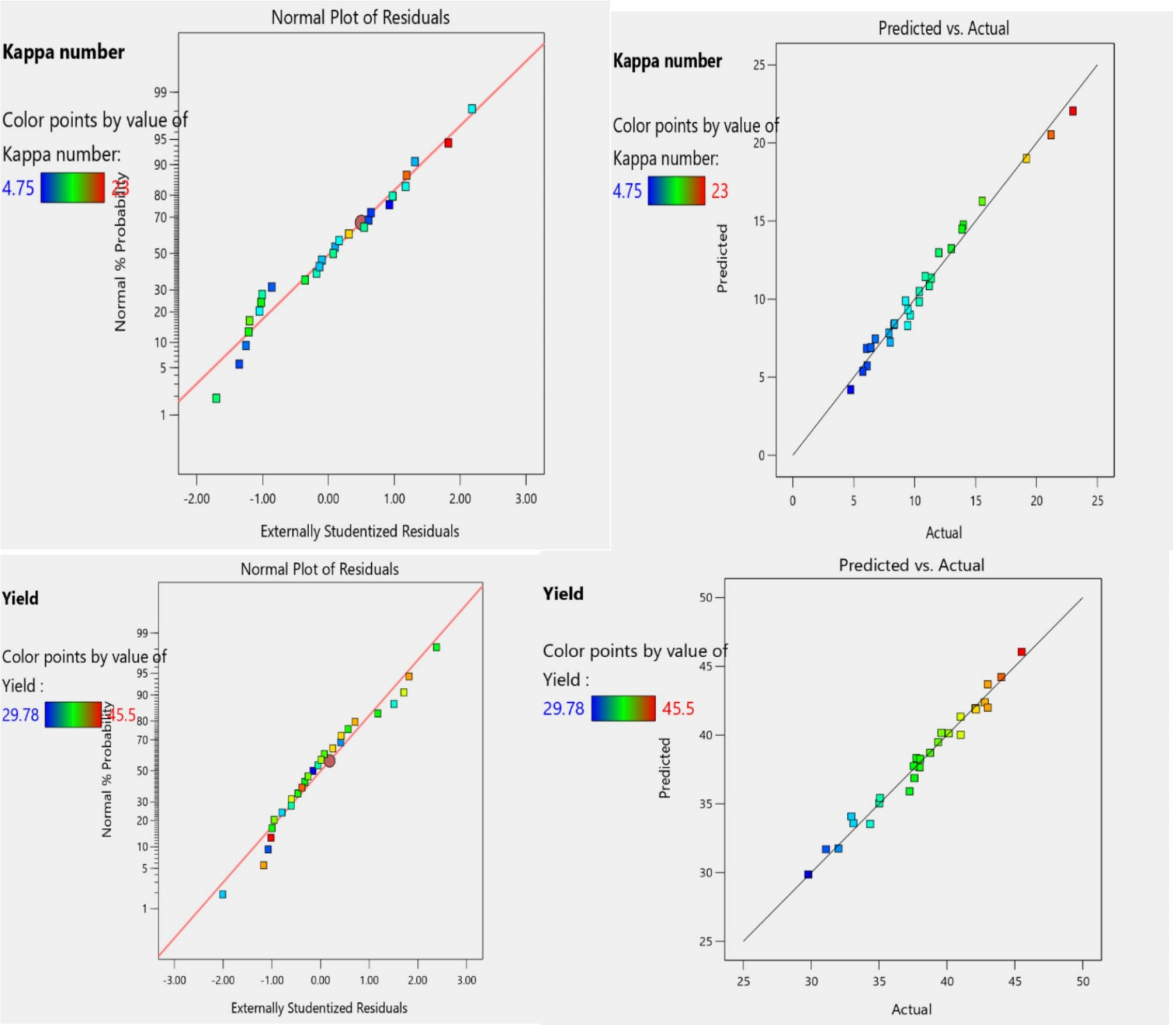
Diagnostic statistics plot.

**Fig. 6:**
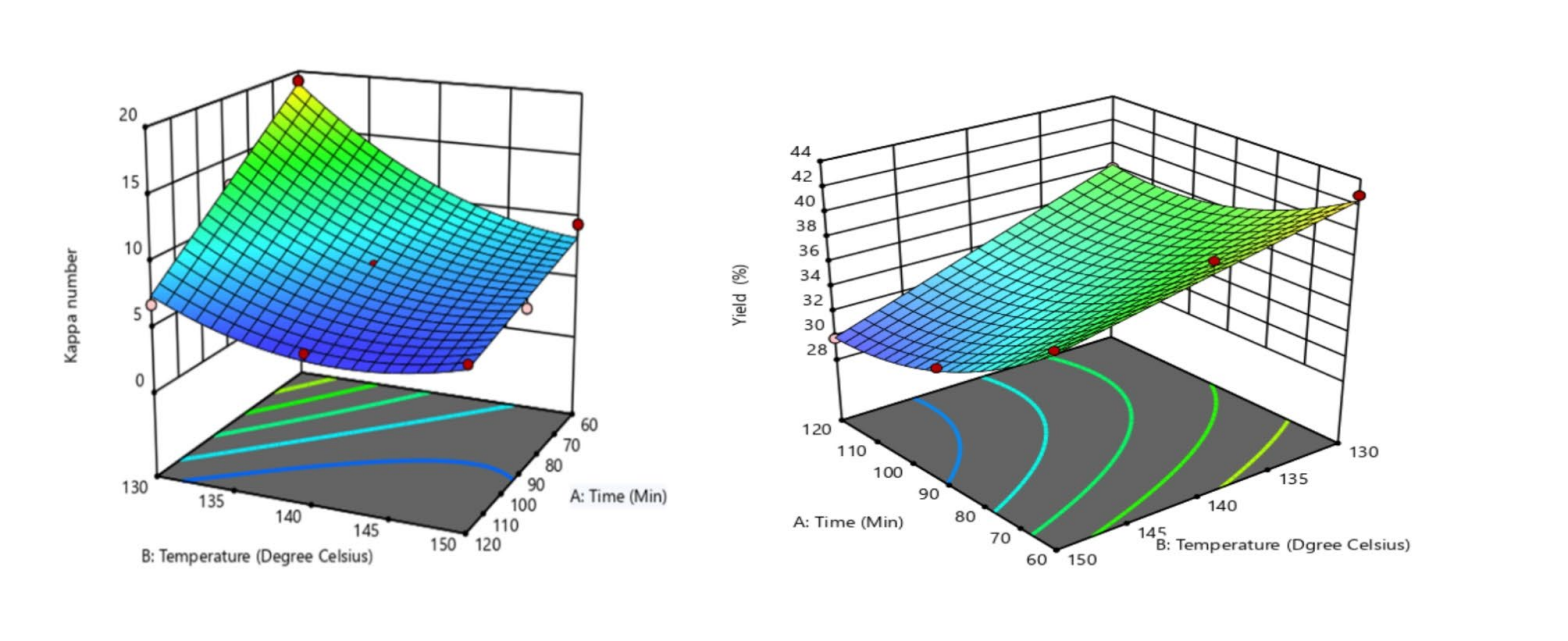
Interaction effect of temperature and time in yield and Kappa number.

The regression analysis was utilized to show the link between the percentage yield and the factors. The primary interaction effects of the variables can be investigated using these charts Figs. 5 and 6, shows that the combined effect of the cooking time and temperature on the yield and Kappa number. When the temperature is increase, the Kappa number increases to a certain level with a decrease of yield of pulp. As the quality of pulp increase with a certain value other response Kappa number is minimum.

As shown in Fig. 6, in pulp production from flax straw, increasing time and temperature lowers the Kappa number, enhancing lignin removal but reducing yield. Higher temperatures and longer cooking times result in better delignification but cause greater material degradation, leading to lower yield. Optimization balances lignin removal with maximizing pulp output.

### Optimization

Model validation or experimental confirmation is the final step in the optimization process using the full factorial design model. In this study, the optimum value of yield and Kappa number was determined using design expert 13. In the software optimization step, the requested goal for each operational variable (cooking time, temperature and active alkaline) was chosen “within the range”, while the response (yield) was placed in “maximum” and Kappa number in minimum as shown in the Table 9. The mean percentage of yield obtained by triplicate experiments is 40.56% as Table 9 which is not significantly different from the predicted value of 41.362% yield and the Kappa number obtained from experiment 10.45 is similar to the predicted value at the optimal conditions are time (120 min), active alkaline (10%), and temperature (131.42℃).

**Table 9:** Result of optimization and model validation.

Number	Cooking time(min)	Temperature (℃)	Active alkaline (%)	Yield (%)	Kappa number
Predicated	120.000	131.420	10.000	41.362	9.800
Experimental	120.000	131.420	10.00	40.560	10.450

**Fig. 7:**
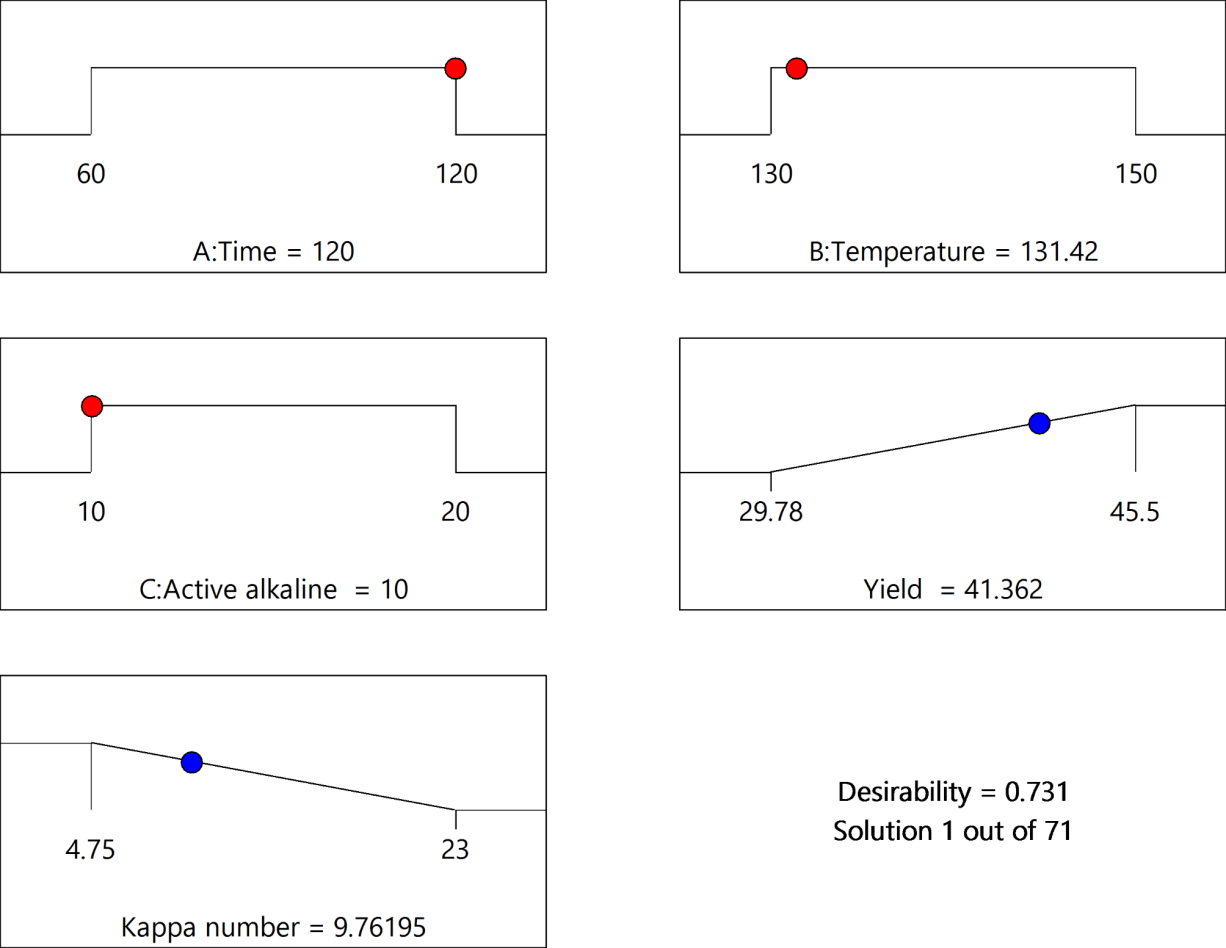
Numerical optimization.

Therefore, as shown in the Fig. 7, the model was valid with desirability 00.731 and capable of predicting the maximum pulp yield numerical optimization can be taken as an optimal value because the predicted value was close enough to the experimental value. In summary, this study shows that active alkaline, temperature, and time could be used for the optimization of pulp produced from flax straw and the yield could be optimized by tuning the concentration of active alkaline, temperature, and time parameters.

### Properties of paper

As a result of the lab, the properties of the paper include three parameters which are tearing, bursting strength and tensile strength. The result of paper product from flax straw compare to the standard used at Wonji paper factor. The standard compares to the base weight of 60 g which means the initial Wight of produced paper in Table 10.

**Table 10:** Properties of paper.

Paper type	Basis weight (g/m^2^)	Burst index kPa	Tear index (mN*m^2^/g)	Breaking length (km)	Ash content (%)	Moisture content (%)
Paper from flax straw (current study)	60	1.40	100	5.20	7.20	6.9
White bond( standard in Wonji paper factory)	60	1.20	65	4.00	6–10	6–8

From Table 10: basis weight of paper for both papers from flax straw and white bond standard in Wonji paper factor used are 60 (g/m^2^). So, with similar basis weight, the other parameter of paper compares to the standard one. The first one is the burst of paper from flax straw is 1.4 kPa greater than the standard value of the white bond (the minimum value is 1.2 kPa). The paper from flax straw is good bursting strength and fulfils the properties of paper regarding bursting strength. Other properties of paper tearing factor, the tearing factor of paper from flax straw is 100mN*m^2^/g which is greater than that of the standard. The breaking length of the paper from flax straw is 5200 m this one is also above the standard. Finally, the basic properties of paper are ash and moisture content with the interval of the standard. Generally, the paper from flax straw has fulfilled all parameters/ properties of paper. This indicated that the production of paper from agricultural waste is important for fulfilling the demand for paper in our country (Ethiopia) and reducing the foreign currency used for imported pulp from the aboard. Even if we used flax for the production of paper there are sides important for the production of linseed for production and extraction of oil the main product as the waste used for paper production.

**Table 11:** Control properties of paper from flax straw.

Paper Properties	Measure Unit	Current Study	Standard Value
Opacity	% (Percentage)	92%	High opacity (85-95%) (Standard Value)
Brightness	% ISO (ISO Brightness)	86%	High brightness (80-90%)
Tensile Strength	N/m (Newtons per meter)	75 N/m	Strong paper (70–80 N/m)

### Paper from flax straw control properties

The results indicate from Table 11 that the paper derived from flax straw exhibits excellent quality across key parameters. With an opacity of 92%, the paper is sufficiently opaque, making it ideal for printing and writing applications where minimal show-through is desired. The brightness level of 86% ISO suggests that the paper has a high degree of whiteness, contributing to sharp contrast and clear visibility of printed text or images. The tensile strength of 75 N/m and burst strength of 320 kPa reflect the paper’s robustness, ensuring durability and resistance to tearing under stress. Collectively, these properties demonstrate that flax straw can be a viable source for producing high-quality paper, suitable for both every day and specialized uses.

## Conclusions

The result from the experiment of proximate properties of flax straw contained ash content and extractive is 4.13 and 5.21 respectively this indicated that the lower ash content means the lower mineral content this range under the standard of biomass proximate properties and preferable for pulp production and used as input for paper production. Chemical compositions get from the experiment, the cellulose content of flax straw was found to be 51.34%, which is satisfactory for pulp production. And it was also found that the flax straw contained low amounts of extractives and ash due to the presence of low silica content. It was found that the yield and Kappa number of flax straw pulp were influenced by pulping variables such as concentrations of active alkali, cooking temperature and time. Pulp yield was slightly influenced by these process variables and a slight decrease in pulp yield was observed upon increasing the levels from the bottom to the next. But a rapid fall was observed in the case of Kappa numbers when increasing levels of process variables from the bottom to the next and most of the delignification processes were carried out during this period. Generally, both the pulp yield and Kappa numbers were inversely related to the processing variables.

## Author declaration

We wish to confirm that there are no known conflicts of interest associated with this publication and there has been no significant financial support for this work that could have influenced its outcome. We confirm that the manuscript has been read and approved by all named authors and there are no other persons who satisfied the criteria for authorship and are not listed.

## Data Availability

Data available from the corresponding author on reasonable request.
